# Tuberculosis control in Ethiopian prisons: A forgotten front in the end TB Strategy

**DOI:** 10.1371/journal.pntd.0014048

**Published:** 2026-03-04

**Authors:** Iris Klaasse, Delesa Wolde Feyisa, Batri Reshu, Alexandra Asboeck, Kumsa Gudeta Aleba, Fabian Schlumberger, Alfenur Abu Kufa, Anil Fastenau

**Affiliations:** 1 Marie Adelaide Leprosy Center (MALC), Karachi, Pakistan; 2 Justice for All-Prison Fellowship Ethiopia (JFA-PFE), Addis Ababa, Ethiopia; 3 German Leprosy and Tuberculosis Relief Association (GLRA), Addis Ababa, Ethiopia; 4 Department of Global Health, Institute of Public Health and Nursing Research, University of Bremen, Bremen, Germany; 5 German Leprosy and Tuberculosis Relief Association (GLRA/DAHW), HQ, Wuerzburg, Germany; Johns Hopkins University, UNITED STATES OF AMERICA

## Background

Tuberculosis (TB) continues to be one of the most devastating infectious diseases worldwide, with more than 10 million new TB cases and 1.5 million TB-related deaths reported annually [1]. Despite being preventable and curable, TB remains a leading cause of antimicrobial resistance (AMR)-related mortality, accounting for nearly one-quarter of all AMR deaths globally [2]. Low- and middle-income countries, including Ethiopia, shoulder the greatest TB burden worldwide [1,3].

Correctional facilities, especially prisons, constitute one of the most neglected yet pivotal settings for tuberculosis control and prevention [4]. The combination of overcrowding, inadequate ventilation, poor nutrition, lack of access to clean water, delayed case detection, and limited access to preventive interventions makes the prevention of *Mycobacterium tuberculosis* transmission particularly challenging [4]. Consequently, incarcerated populations remain disproportionately burdened by TB infection and disease, representing a critical but under-addressed component of national and global TB elimination strategies [5]. Thus, prisons represent one of the most overlooked yet critical environments for TB control and prevention as inmates are a marginalized population at exceptionally high risk of developing and transmitting the disease. Globally, 11 million people are incarcerated, 1.4 million of them in Africa [6], and the burden of TB among incarcerated populations is estimated to be up to ten times higher than in the general population [4,5]. Yet, these settings and the most vulnerable population living in the prisons are often excluded from national TB surveillance and health system planning.

Experiences from multiple correctional facilities across Ethiopia reveal that, despite adherence to national TB guidelines, major systemic barriers continue to undermine effective disease control and prevention. This viewpoint draws on direct observations across multiple correctional facilities in Ethiopia and interviews with prison health professionals and individuals undergoing TB treatment while incarcerated.

## The overlooked epidemic in Ethiopian prisons

Ethiopia is among the 30 high TB burden countries [3], with an incidence of approximately 146 per 100,000 in 2023 [7]. While national TB incidence and mortality rates have steadily declined in recent years, the burden within correctional facilities remains disproportionately high [8]. According to the Ministry of Health mid-term review, prison notification rates reached 867 per 100,000 population in 2025, approximately six times the national incidence rate [8]. Many Ethiopian prisons operate far beyond their intended capacity, with inmates confined in overcrowded, poorly ventilated, and unhygienic environments, creating ideal conditions for TB transmission and persistence [4].

Drug-resistant TB remains a concern. Nationally, the prevalence of RR/MDR-TB among new TB cases was 1.13% in 2025, reflecting sustained but persistent transmission [8]. Although comprehensive molecular transmission studies within prisons remain limited, periodic community-based screening campaigns conducted in 53 prison facilities detected substantial numbers of bacteriologically confirmed TB and RR-TB cases, underscoring ongoing active transmission in these high-density environments [8]. Furthermore, limited routine implementation of systematic latent TB infection (LTBI) screening in prisons constrains understanding of transmission dynamics and progression from infection to active disease.

Experiences from Yirgalem prison in southern Ethiopia illustrate how these structural factors shape daily clinical realities. A health professional described an inmate undergoing TB treatment whose therapy was interrupted during transfer to police custody: “He had started the medication several months ago and stopped taking it after being taken to the police station.” In overcrowded cells housing far more inmates than intended, such interruptions, combined with close physical proximity and limited isolation space, increase the risk of ongoing transmission. Health staff further reported that inadequate food provision frequently leads to malnutrition among TB patients, undermining treatment outcomes despite the availability of medication.

Interviews with incarcerated individuals further highlighted the daily realities behind these interruptions. One prisoner described persistent coughing and progressive weight loss while waiting for treatment to resume, explaining that “the medicine was strong, but the food was not enough.” Such accounts illustrate how treatment gaps and malnutrition intersect within overcrowded environments.

While national TB programs provide medications and periodic screening, broader determinants of TB transmission, such as nutrition, water, sanitation, and hygiene (WASH), and mental health, remain inadequately addressed. This neglect reflects broader systemic challenges seen across sub-Saharan Africa, where prison health often falls outside mainstream public health policy and accountability mechanisms.

## Major challenges identified for TB control and prevention in Ethiopian prisons

### 1. Overcrowding and poor ventilation

Overcrowding remains one of the greatest challenges to TB control and prevention in prisons [9]. In Ethiopian prisons, inmates are often crammed into poorly ventilated rooms. The lack of cross-ventilation, mechanical airflow, and adequate isolation areas makes infection control nearly impossible [9]. Isolation rooms for active TB patients are often few, substandard, and built from corrugated metal sheets that expose inmates to extreme heat and cold. The absence of isolation facilities and limited infection-control measures heightens transmission risk.

### 2. Inadequate water, sanitation, and hygiene (WASH)

Many prisons face chronic shortages of clean water and unreliable electricity, disrupting hygiene and infection control. Poor sanitation contributes not only to TB but also to other infectious diseases such as diarrhea and respiratory infections [10]. Inadequate WASH infrastructure reflects deep inequities in access to essential services between the incarcerated and the general population.

### 3. Malnutrition and food insecurity

Nutrition plays a critical role in TB susceptibility and treatment success [11]. The daily food budget in an Ethiopian prison is grossly insufficient. Health professionals estimate that roughly one-third of TB patients experience moderate acute malnutrition, while a small proportion are affected by severe malnutrition. This weakens immunity, delays recovery, and increases relapse risk of TB [12]. Sustainable nutrition interventions, such as poultry or livestock farming within prisons, could improve both health and rehabilitation outcomes for the people affected by TB residing in prisons.

### 4. Weak referral systems and treatment continuity

Interruptions in TB treatment frequently occur during transfers from police custody to prison, or when inmates are released without linkage to follow-up care. The lack of an integrated health information and referral system between prisons, police stations, and regional health facilities leads to treatment default and heightened risk of multidrug-resistant TB (MDR-TB). In addition, laboratory capacity within prisons and the broader prison-referral network remains inadequate. The lack of standardized intake and periodic screening further delays case detection. These diagnostic and operational gaps contribute to preventable transmission and increase the risk of drug-resistant TB emerging within correctional settings.

### 5. Limited staff training and motivation

Health professionals working in Ethiopian prisons frequently lack specialized TB training. Health staff operate in small, poorly ventilated TB clinics that endanger their own health. Frequent staff turnover, low pay, and lack of psychosocial support contribute to low motivation. Without continued capacity building and incentives, sustaining quality TB care remains difficult.

### 6. Stigma and cultural barriers

Stigma surrounding TB persists despite educational efforts [13]. Some inmates conceal their illness for fear of exclusion, while cultural beliefs, such as reliance on holy water or fasting during treatment, undermine adherence. Although awareness campaigns have improved acceptance, TB stigma continues to hinder timely diagnosis and treatment completion. Health workers described fear of social exclusion and isolation as reasons why some inmates conceal symptoms, delaying diagnosis and increasing transmission risk. Furthermore, some incarcerated individuals described concealing symptoms due to fear of isolation or discrimination from other inmates. This hesitation to report illness contributes to delays in diagnosis and sustained transmission.

## Systemic implications and human rights dimensions

Prison health is an essential component of the human right to health, as enshrined in the UN Mandela Rules and the WHO’s *Prisons and Health* framework [14]. Yet, conditions in many low-resource prisons in Ethiopia violate these standards. The failure to ensure adequate food, sanitation, and medical care undermines both human dignity and public health goals. The right to health within prisons extends beyond disease treatment, it encompasses living conditions, mental well-being, and rehabilitation opportunities. In alignment with the WHO End TB Strategy, this calls for comprehensive, multisectoral interventions that address the social determinants of health and strengthen TB prevention and care within prisons [15]. Health staff expressed concern that untreated or interrupted cases inside prisons may contribute to transmission beyond prison walls upon release.

## Recommendations and action points

Experiences from Ethiopian prisons reveal practical approaches to improving TB control and prevention in similar contexts across sub-Saharan Africa.

### 1. Strengthen infrastructure and WASH systems

- Ensure reliable access to clean water and electricity through collaboration with local authorities.
- Construct ventilated and standardized TB isolation facilities.
- Leverage Ethiopia’s ongoing rollout of WHO-supported AI-enabled digital chest X-ray systems by formally integrating prisons into the national screening program.
- Integrate WASH improvements (handwashing stations, toilets, waste disposal) into all prison health projects.

### 2. Improve nutrition and livelihoods

- Increase the prison food budget to meet nutritional needs, particularly for TB patients.
- Establish sustainable agricultural programs (e.g., poultry, livestock, or vegetable farming) for nutrition and rehabilitation.

### 3. Address overcrowding through penal reform

- Implement alternative sentencing for non-violent offenders to reduce congestion.
- Expedite construction of new prison facilities and introduce community-based correction models.

### 4. Build health workforce capacity

- Provide regular TB, WASH, and infection-control training for all prison health staff.
- Introduce motivation and retention measures, including regular supervision, recognition programs, and continued education.
- Introduce trained inmate peer educators to support health education, symptom screening, and active case finding.

### 5. Ensure continuity of care and strengthen referral systems

- Develop a standardized national referral system connecting prisons, police stations, and local health facilities.
- Implement digital health records to track treatment progress from pre-trial detention through post-release.
- Institutionalize pre-release counseling and linkage-to-care for all TB patients, especially those with DR-TB.
- Given evidence of MDR-TB within Ethiopian prison populations, all referrals should include drug-resistance testing, timely laboratory confirmation, and adherence support.

### 6. Promote advocacy, research, and policy integration

- Prioritize prison health in the National TB and End TB Strategies.
- Foster partnerships between ministries (Health, Justice, Water, Agriculture) and non-governmental organizations (NGOs).
- Encourage operational research on TB and AMR in correctional facilities to guide policy reform.

## Conclusion

Ethiopian prisons reflect the global neglect of TB in correctional facilities, settings that concentrate vulnerability, poverty, and disease. Yet, it also offers a model for change. The existing collaboration between prison authorities, the Regional Health Bureau, and NGOs demonstrates that progress is possible even in resource-limited settings.

Prisons should not remain blind spots in TB control and prevention. Strengthening health systems within these institutions is both a moral imperative and a public health necessity. TB control in prisons benefits not only inmates but also the wider community by reducing transmission and drug resistance. Integrating prison health into national TB and AMR strategies, investing in infrastructure and workforce development, and ensuring human rights standards are upheld will be essential for achieving the End TB goals. TB control in prisons is, ultimately, TB control for all. No one should be left behind, not even those behind bars.
